# Two- and three-input TALE-based AND logic computation in embryonic stem cells

**DOI:** 10.1093/nar/gkt758

**Published:** 2013-08-27

**Authors:** Florian Lienert, Joseph P. Torella, Jan-Hung Chen, Michael Norsworthy, Ryan R. Richardson, Pamela A. Silver

**Affiliations:** ^1^Department of Systems Biology, Harvard Medical School, Boston, MA 02115, USA, ^2^Department of Molecular and Cellular Biology, Harvard University, Cambridge, MA 02138, USA and ^3^Wyss Institute for Biologically Inspired Engineering, Harvard University, Boston, MA 02115, USA

## Abstract

Biological computing circuits can enhance our ability to control cellular functions and have potential applications in tissue engineering and medical treatments. Transcriptional activator-like effectors (TALEs) represent attractive components of synthetic gene regulatory circuits, as they can be designed *de novo* to target a given DNA sequence. We here demonstrate that TALEs can perform Boolean logic computation in mammalian cells. Using a split-intein protein-splicing strategy, we show that a functional TALE can be reconstituted from two inactive parts, thus generating two-input AND logic computation. We further demonstrate three-piece intein splicing in mammalian cells and use it to perform three-input AND computation. Using methods for random as well as targeted insertion of these relatively large genetic circuits, we show that TALE-based logic circuits are functional when integrated into the genome of mouse embryonic stem cells. Comparing construct variants in the same genomic context, we modulated the strength of the TALE-responsive promoter to improve the output of these circuits. Our work establishes split TALEs as a tool for building logic computation with the potential of controlling expression of endogenous genes or transgenes in response to a combination of cellular signals.

## INTRODUCTION

A major focus of synthetic biology is the design of genetic circuits that program cellular functions in living organisms. The development of such circuits in mammalian cells has the potential to lead to new strategies for cell-based therapies and diagnostics (1–3). Bio-molecular computing systems have been implemented using various components, including recombinases, small RNAs, riboswitches and natural and artificial transcription factors (TFs) (4). Whereas the first synthetic transcriptional networks in eukaryotes relied on well-characterized bacterial TF–promoter pairs, recent work has used zinc finger TFs (5,6). Zinc finger proteins offer the advantage that their DNA binding domain can be designed to recognize specific sites, but the predictability of this engineering process can sometimes be a challenge (7).

Transcriptional activator-like effectors (TALEs) are an alternative class of transcription activators whose DNA binding specificity is more amenable to engineering (8–10). DNA recognition by TALEs is mediated by a protein domain consisting of a variable number of linearly arranged TAL repeats that are, on average, 34 amino acids in length. Two variable amino acids within each TAL repeat determine the specificity towards a single nucleotide in the TALE DNA recognition site. When combined with transcriptional activator or repressor domains, TALEs are able to regulate expression of endogenous genes and transgenes (11–15). Furthermore, Li *et al.* (16) have recently developed TALE hybrids that can be regulated by either addition of exogenous ligands or by endogenous pathways, such as hypoxia signaling or microRNAs.

Given their potential as regulators of gene expression and as parts of synthetic transcriptional networks, we tested whether TALEs can be used to perform AND logic computation in mammalian cells. For this purpose, we used an intein-mediated protein splicing approach. Specifically, we made use of split inteins, which can auto-catalytically *trans*-splice protein fragments to which they are fused (17,18). Using this system, we implemented TALE-based computation in mammalian cells.

For practical applications of bio-computational circuits in cell therapy, it is necessary to stably maintain them in the cell type of interest. Currently, components of newly developed mammalian synthetic transcriptional networks are often expressed from separate plasmids; genomic integration of such circuits therefore necessitates the use of multiple selection markers, and leads to differences in the copy number of individual components (19,20). To circumvent these limitations, we assembled the TALE-based logic circuits as single DNA constructs and show that they maintain their functionality. We also demonstrate that they can perform AND computation when integrated into the genome of mouse embryonic stem (ES) cells. Using a site-directed insertion approach further allowed us to optimize circuit variants in the same genomic environment. Our finding that genomically integrated TALE-based circuits are functional in pluripotent ES cells suggests that they could be used as sensors in cell therapy applications or for directing *in vitro* differentiation in tissue engineering.

## MATERIALS AND METHODS

### Recombinant DNA constructs

From a list of 20-bp-long potential TALE binding sites that are orthogonal to gene promoters in the human genome (13), we selected one that is also orthogonal to mouse gene promoters. The corresponding TALE, TAL118, was assembled using a Golden-Gate cloning scheme (13). The split intein fragments, which have been described previously (5), were fused to split TALE fragments using polymerase chain reaction and Alw26I Type-IIS restriction enzyme methods. Expression constructs were generated by combining BioBrick subparts using Biobrick assembly (21,22). For testing circuits with each part on a separate plasmid, DNA fragments were cloned between NheI and NotI sites of vector pCDNA5ins (5) and reporter fragments between SpeI and NotI sites of pCDNA5/FRT/TO for mammalian expression (Invitrogen). For assembly of circuits on a single DNA construct, an isothermal assembly-based hierarchal cloning scheme was used (Torella *et al.*, submitted). Circuits were assembled on two modified versions of vector pETcoco-1 (Novagen); pDestRmceBAC, which includes two inverted loxP sites around the assembled circuit, and pDestPBBAC, which contains a Blasticidin resistance marker, two inverted terminal repeats and a PiggyBac transposase (from vector pHULK, DNA2.0). Sequence parts are listed in Supplementary Table S1.

### Cell culture

The human osteosarcoma-derived epithelial cell line U-2 OS (ATCC no. HTB-96) was maintained and transfected as previously described (5). TC-1 ES cells (background 129S6/SvEvTac) containing a recombinase-mediated cassette exchange (RMCE) target site were cultured as previously described (23). Transient transfection of ES cells was performed in six-well plates seeded with ∼750 000 cells using 10 μl of Lipofectamine 2000 reagent (Invitrogen) with 3 μg total DNA per well. A summary of plasmid amounts used for transfections can be found in Supplementary Table S2. For low-copy random integrations using the PiggyBac system, ES cells were transfected using Lipofectamine and selected with 10 μg/ml Blasticidin (Invitrogen) for 7 days starting 48 h post-transfection. ES cell lines with single-copy integration in the beta-globin locus were obtained by RMCE, as previously described (23). In brief, 4 × 10^6^ ES cells were electroporated with 25 µg of target vector and 15 µg of the Cre Recombinase expression plasmid pIC-Cre. Selection with 3 µM ganciclovir (Roche) was started 48 h post electroporation and continued for 10–12 days. Resistant clones were tested for successful insertion by polymerase chain reaction. Only clones that integrated the target construct in the same orientation were compared.

### Microscopy

Microscopy of transiently transfected U-2 OS was performed on live cells 48 h post-transfection. Cells were imaged by a Nikon TE-2000 microscope with a 20× PlanFluor NA = 0.5, DIC M/N2 objective and an ORCA-ER charge-coupled device camera. Data collection and processing were performed using Metamorph 7.0 software (Molecular Devices). All images within a given experimental set were collected using the same exposure times, averaged over three frames and underwent identical processing.

### Flow cytometry

Approximately 50 000–100 000 live cells were analyzed using an LSRII cell analyzer (BD Biosciences). Cells were trypsinized, pelleted and resuspended in Dulbecco’s phosphate-buffered saline containing 0.1% fetal bovine serum. For transient transfection experiments, output was assayed 48 h post-transfection. To control for variations in transfection efficiency among different samples, we gated for cells that are positive for mCherry (mCh), which is expressed from a constitutive cytomegalovirus (CMV) promoter in all tested constructs. Within these mCh-positive cells, we determined the percentage of AmCyan fluorescent protein (CFP)-positive cells (% CFP+ in mCh+). An example of the raw data and gating procedure is given in Supplementary Figure S1. For testing the background activity of the reporter plasmid, it was co-transfected with a mCh-tagged off-target TALE. The off-target TALE, TAL248, binds to TATACTATCCAATCCAACTT (13). Flow cytometry results for all experiments are listed in Supplementary Table S3.

## RESULTS

### Split intein-mediated splicing of a TALE enables two-input logic computation

**Figure 1.**
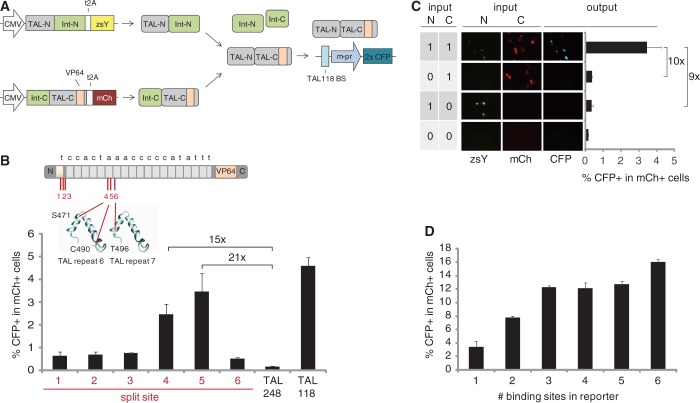
Engineering of a split TALE-based AND circuit. (**A**) Schematic of the split TALE reconstitution process. TAL-N:Int-N and Int-C:TAL-C fragments are expressed from CMV expression plasmids. The two split TAL–intein fragments dimerize and undergo protein splicing to reconstitute the full TALE, which binds and activates a CFP reporter containing a binding site for TAL118 upstream of a HSV minimal promoter (m-pr). TAL118 induces expression through a transcriptional activator domain VP64 (24). The N- and C-TAL:Int fragments contain nuclear localization signals and are tagged with co-translationally cleaved t2A:zsYellow and t2A:mCherry, respectively. (**B**) Characterization of the 6 split TALE–intein pairs assayed by transient transfection in U-2 OS cells. Reporter activity is indicated by the percentage of CFP positive in all mCh-positive cells, as measured by flow cytometry. As a control, the reporter plasmid was transfected with TAL118 or the off-target TAL248 (with both constructs containing a mCh tag). The inlay illustrates the positions of tested split sites in TAL118, with its cognate binding site shown above (from 5′ to 3′). The structural model illustrates the positions of split sites 4 to 6 in TAL repeat 6 and 7 (25). (**C**) Fluorescence microscopy images and flow cytometry results (bar graph) for the TAL118_s5 fragments transfected separately or in combination illustrating the AND gate behavior of the circuit. To control for transfection efficiency, input 10 and 00 were co-transfected with a construct expressing mCh. (**D**) Co-transfection of TAL118_s5 AND circuit fragments with reporter plasmids containing 1–6 binding sites for TAL118. Error bars indicate standard deviation from three biological replicates.

We used intein-mediated protein splicing to implement Boolean logic AND gates (5). Our system makes use of TALE fragments incapable of activating transcription on their own; when both are present and correctly spliced, however, they reconstitute an active TALE capable of activating transcription, thereby creating a two-input AND gate in cells (Figure 1A).

As we desired to use a synthetic TALE with minimal cross-reactivity to the endogenous genome, we first computationally designed a TALE to have no cognate binding sites or predicted off-target binding sites (up to three mismatches) in the 1000-bp promoter regions of mouse and human genomes (13). We then characterized the ability of this TALE, TAL118, to activate expression of CFP from a promoter containing its cognate binding site. Using flow cytometry, we confirmed that TAL118 is able to induce reporter transcription in U2-OS osteosarcoma cells (Supplementary Figures S1 and S2).

We next determined the optimal amino acid residues at which to divide TAL118 for use in a split-intein protein splicing strategy. We chose three split sites in the protein domain that is essential for binding the 5′ thymine in the TALE binding site and three sites in TAL repeats 6 and 7 of the DNA binding domain (Figure 1B). Based on these split points, we created six pairs of TAL118 split proteins, with each pair containing an amino- (N-) and a carboxy- (C-) terminal fragment fused to the appropriate part of a split dnaB mini-intein from *Rhodothermus marinus* (5). We cloned these fragments into expression vectors and co-transfected them with a reporter containing a TAL118 binding site upstream of a minimal herpes simplex virus type 1 (HSV) promoter and a CFP gene (Figure 1A). We assayed for reporter activation using flow cytometry and controlled for transfection efficiency by calculating the percentage of CFP-expressing cells within the population of successfully transfected cells (based on the expression of mCh). These values, as well as the mean CFP signal within mCh-expressing cells, can be found in Supplementary Table S3. Two of six TAL118 split pairs showed a greater than 10-fold induction in the number of CFP+ cells relative to an off-target TALE activator, with both pairs originating from split sites in TAL repeat 6 (Figure 1B). The TAL118 fragment pair originating from split site 5 (TAL118_s5) led to the highest induction (21-fold) and was used throughout the remaining experiments. Importantly, transfection of the TAL118_s5 fragment pair led to a 10-fold and 9-fold higher induction in the number of CFP+ cells relative to either the N- or C-fragments alone, confirming the AND gate behavior of this circuit (Figure 1C). Furthermore, circuit output increased when additional TAL118 DNA binding sites were added to the reporter, with inclusion of six binding sites leading to 16% CFP+ cells (Figure 1D). Taken together, these results demonstrate the feasibility of building an AND gate based on a split TALE, and show that TALE-based synthetic transcriptional activator proteins can be used for bio-molecular computation in mammalian cells.

### A three-piece intein enables TALE-based three-input logic AND computation

**Figure 2.**
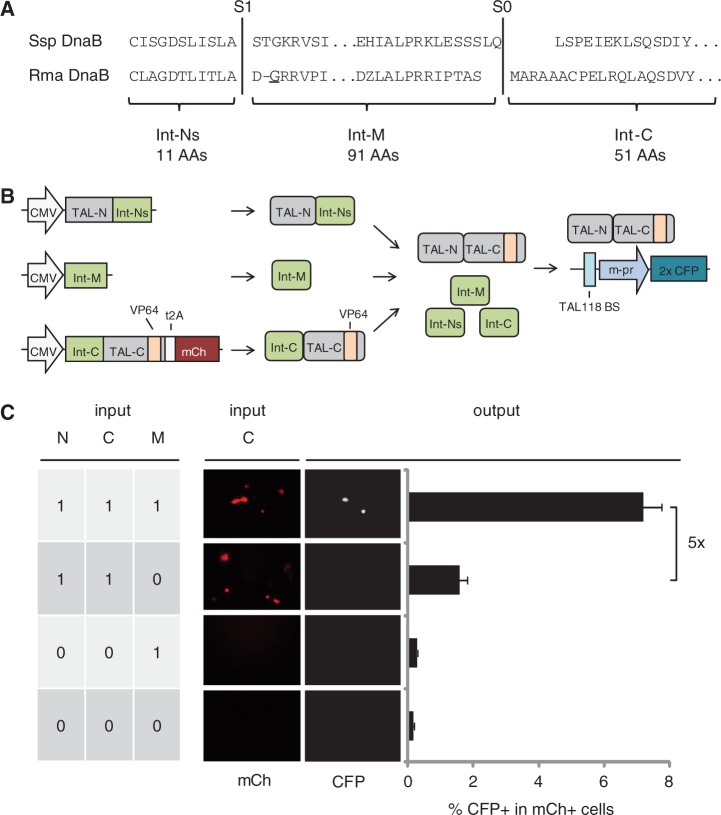
Engineering of a split TALE-based three-input AND circuit. (**A**) Part of the amino acid sequence of the *Ssp* (top) and *Rma* (bottom) DnaB mini-intein is shown. S1 and S0 correspond to split sites in the *Ssp* three-piece intein as reported by Sun *et al.* (26). Split sites in the *Rma* DnaB mini-intein were chosen at homologous positions and a nuclear localization signal was added to fragment Int-M. The numbers at the bottom indicate the length of the resulting intein fragments in numbers of amino acids (AAs). The glycine highlighted with an underline indicates the stop codon location in circuit 3i-Ctrl2 (compare with Figure 3C). (**B**) Schematic of the three-input circuit illustrating that reconstitution of the split TALE depends on a three-piece intein. (**C**) Fluorescence microscopy images and flow cytometry results of the three-piece intein TAL118_s5 fragments transfected separately or in combination illustrating the AND gate behavior of the circuit. To control for transfection efficiency, input 001 and 000 were co-transfected with a construct expressing mCh. Reporter activity is indicated by the percentage of CFP positive in all mCh-positive cells, as measured by flow cytometry. Error bars indicate standard deviation from three biological replicates.

A three-piece intein, originating from a dnaB mini-intein from *Synechocystis sp.*, has previously been shown to induce protein *trans*-splicing in *Escherichia coli* (26). We investigated whether we could use such a three-piece intein to make the transcriptional AND gate circuit dependent on expression of an additional peptide and thereby extend it to three inputs. However, as the *Ssp* dnaB intein is expected to show low splicing efficiency in mammalian cells (27), we chose homologous split sites in the dnaB mini-intein from *R. marinus* (Figure 2A). We fused the N- and C-parts (Int-Ns and Int-C) of this three-piece intein to the corresponding TAL118_s5 fragments (Figure 2B). Co-transfection of these two parts with the middle part of the three-piece intein (int-M) led to a 43-fold induction in the number of CFP+ cells relative to an off-target control (Figure 2C), and to a 5-fold induction relative to a control lacking int-M, showing that high transcriptional output depends on the presence of all three input parts. These results demonstrate that three-piece intein-mediated splicing is functional in mammalian cells and that it can be applied to build a TALE-based transcriptional circuit that performs three-input AND computation.

### Expression of AND gates from single plasmids and optimization of reporter output

**Figure 3.**
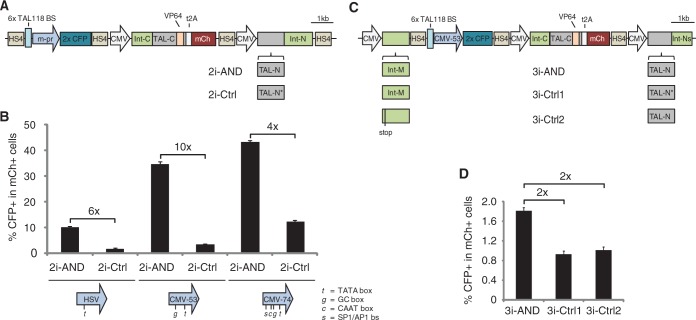
Expression of two- and three-input AND gate circuits from a single plasmid. (**A**) Circuit 2i-AND reconstitutes TAL118, which binds the reporter. Four TAL repeats were exchanged in the N-input of 2i-Ctrl (TAL-N*), leading to reconstitution of a TALE designed to recognize a binding site that differs by 4 bp from the TAL118 binding site (tcataaaaacccccatattt). (**B**) Characterization of 2i-AND and 2i-Ctrl circuits by transient transfection in ES cells. For both circuits, variants with three different minimal promoters (m-pr) in the reporter part were tested (HSV, CMV-53 and CMV-74). The locations of regulatory elements in these promoter regions are indicated at the bottom of the promoter symbols. (**C**) Circuit 3i-AND reconstitutes TAL118, which binds the CMV-53 reporter. 3i-Ctrl1 contains the same TAL-N* part as 2i-Ctrl. 3i-Ctrl2 differs from 3i-AND by containing a premature stop codon in the intein middle part (compare with Figure 2A). (**D**) Characterization of 3i-AND and its control circuits in ES cells. Reporter activity in (B) and (D) is indicated by the percentage of CFP positive in all mCh-positive cells, as measured by flow cytometry. Error bars indicate standard deviation from three biological replicates.

The performance of synthetic transcriptional circuits whose parts are expressed from separate plasmids can be hampered by noise stemming from variations in transfection efficiency. Assembly of a circuit as a single DNA construct circumvents this problem by ensuring a 1:1 ratio of its subparts and also facilitates genomic integration, which is necessary in many practical applications. For these reasons, we cloned all components of the TAL118_s5 two-input AND circuit onto a single DNA construct. To do so, we used a modular, isothermal assembly-based cloning method that allows efficient construction of DNA parts in a bacterial artificial chromosome. Using this system, we generated construct 2i-AND consisting of a TAL118-responsive CFP reporter upstream of the two constitutively-expressed C- and N-input parts of TAL118_s5 (Figure 3A). To decrease the likelihood of transcriptional interference between each part, we separated the individual gene components by inserting HS4 insulator sequences (28). We also made a control circuit (2i-Ctrl) by replacing four TAL repeats in the N-fragment (TAL-N) of 2i-AND. 2i-Ctrl reconstitutes a TALE with a DNA binding domain that is unable to recognize the TAL118 binding site in its reporter part. As all other parts in these two circuits are identical, 2i-Ctrl provides a control for TAL118-independent activation of the reporter in 2i-AND. Flow cytometry revealed that transient transfection of 2i-AND leads to a 6-fold induction in the number of CFP+ cells relative to 2i-Ctrl in ES cells (Figure 3B). This finding confirms that transcriptional output from 2i-AND is dependent on TAL118 binding and shows that the two-input AND gate is functional when expressed from a single plasmid.

In practical applications of the 2i-AND circuit, promoters of endogenous genes might drive expression of its input fragments. As such promoters are often weaker than the viral promoters used here, we sought to optimize transcriptional output of 2i-AND by modifying its reporter part. We replaced its minimal HSV promoter with either of two minimal CMV promoter variants, CMV-53 and CMV-74, which contain more regulatory sequences than the single TATA box present in the HSV promoter (29). We found that transfection of the CMV-53-containing 2i-AND circuit led to a 10-fold induction in the number of CFP+ cells relative to its corresponding control circuit (Figure 3B), and to a 3-fold induction relative to the HSV-containing 2i-AND, demonstrating that a stronger core promoter can enhance transcriptional output. Although inclusion of CMV-74 in 2i-AND led to similar levels of CFP+ cells (4-fold induction relative to the HSV-containing circuit), this circuit showed an only 4-fold increase in CFP+ cells relative to its corresponding control circuit due to the increased background activity of the CMV-74 promoter (Figure 3B).

Having enhanced the transcriptional output of 2i-AND, we tested whether the three-input AND circuit would be functional when arranged on a single plasmid. For this purpose, we assembled the CMV-53 reporter and the three input parts into circuit 3i-AND (Figure 3C). We also made two control constructs; one containing an altered TAL-N part (3i-Ctrl1) and one containing a premature stop codon in the middle part of the intein (3i-Ctrl2) (Figures 2A and 3C). Transfection of these constructs revealed that 3i-AND shows a 2-fold higher induction of CFP positive cells than the two control circuits, demonstrating that the three-input TAL118 AND circuit is functional when expressed from a single plasmid. Taken together, these experiments show that both the two- and three-input TAL118 AND circuit remain functional when expressed from a single DNA construct and that exchanging the minimal promoter in the reporter can enhance transcriptional output.

### Genomic integration of circuits

Practical applications of bio-computation may require stable maintenance of genetic circuits in the cell type of interest. We therefore tested whether the TAL118 AND circuit would work when stably integrated into the genome. For this purpose, we performed genomic integration in mouse ES cells, a primary cell type that has the potential to be differentiated into any cell type of interest.

**Figure 4.**
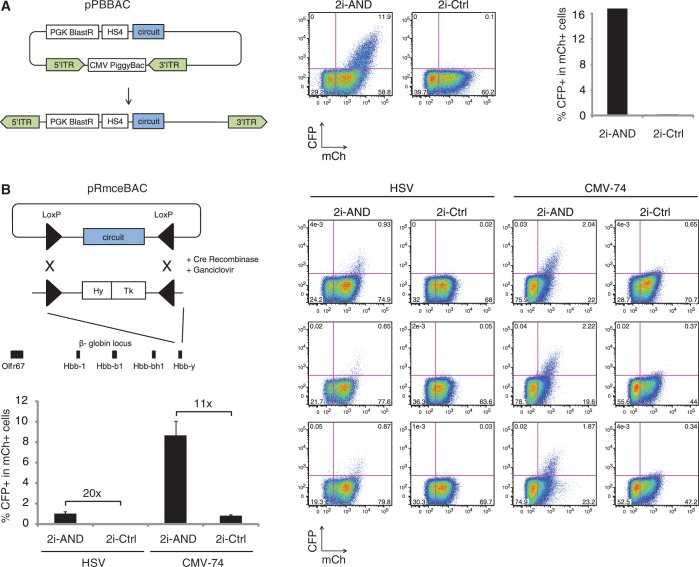
Characterization of genomically integrated AND circuits. (**A**) The diagram depicts the pPBBAC system for low-copy, random genomic integration. On transfection, the PiggyBac transposase integrates the DNA circuit located in between two inverted terminal repeat sequences (ITR). A PGK promoter-driven Blasticidin resistance marker allows selection of stable integrants. The flow cytometry scatter plots show CFP and mCh signals after PiggyBac-mediated integration of 2i-AND (left) and 2i-Ctrl (right) in ES cells. The numbers indicate percentage of cells in each quadrant gate. (**B**) The diagram depicts the pRmceBAC system for single-copy genomic integration. Circuits are integrated by Cre recombinase in a target site in the beta-globin locus that consists of two inverted loxP elements (triangles) flanking a fusion of a hygromycin-resistance (Hy) and a ganciclovir-sensitivity gene (Tk). The flow cytometry scatter plots show CFP and mCh signals of ES cells with integrated 2i-AND and 2i-Ctrl circuits, each containing either promoter variant HSV or CMV-74. Each row displays results for an independent cell clone. The numbers indicate percentage of cells in each quadrant gate. The bar graph shows the percentage of CFP positive in all mCh-positive cells. Error bars indicate standard deviation from three independent cell clones.

First, we used a system that enables efficient genomic integration at a low copy number. For this purpose, we inserted our circuits between two inverted terminal repeat sequences on a plasmid that expresses PiggyBac transposase, which on transfection will move them into chromosomal TTAA sites (30) (Figure 4A). Using this method, we integrated the 2i-AND circuit containing the HSV minimal promoter and its corresponding control 2i-Ctrl to generate a polyclonal population of cells, presumably containing the circuits at variable copy numbers and insertion sites. Integration of 2i-AND resulted in a high proportion of cells that induce reporter activity, with 16.8% of cells positive for mCh also positive for CFP expression. In case of the 2i-Ctrl circuit, the proportion of mCh+ cells that were also CFP+ was only 0.2%, suggesting that 2i-AND has little background activity in a genomic context. These results show that the two-input AND circuit is functional in a genomic environment.

We next tested whether 2i-AND would also be active when integrated into the genome as a single copy. For this purpose, we used a stem cell line with a target site in the beta-globin locus, which allows efficient integration of DNA constructs by RMCE (23) (Figure 4B). Importantly, this integration method enables one to compare different circuits in the same genomic environment. Again, the control circuit 2i-Ctrl showed very low reporter activity when integrated in this genomic target site. Conversely, the 2i-AND circuit activated CFP expression leading to a 20-fold induction in the number of CFP+ cells relative to the control circuit. These results demonstrate that the 2i-AND circuit is functional when integrated as a single copy.

By integrating constructs in the same genomic target site, we found that the promoter variant CMV-74 enhanced the transcriptional output of 2i-AND in a genomic environment. Inclusion of CMV-74 increased the population of CFP+ cells by 8-fold relative to the HSV-containing circuit (Figure 4B). Relative to its control circuit, it induced an 11-fold higher number of CFP+ cells, markedly greater than that achieved in our transient transfection experiments (4-fold, Figure 3B). This higher relative induction is caused by the lower background activity of CMV-74 in the genomic target site. Of note, we observed that the genomically inserted CMV-74-containing 2i-AND circuit showed lower expression of mCh-tagged TAL-C:int-C than its corresponding control circuit (21.6% versus 54.0% mCh+ cells). This might be caused by insufficient insulation of the TAL-C:int-C expression cassette from the upstream CFP reporter and resulting transcriptional interference.

In contrast to 2i-AND, similar genomic integration of 3i-AND did not lead to induction of CFP. This is presumably due to lower expression of its composite parts, which may decrease splicing efficiency.

These results demonstrate that a TALE-based transcriptional circuit is able to perform two-input AND computation as a single-copy integrant in ES cells. Site-directed integration allowed comparison of circuit variants in the same genomic context and helped us to improve transcriptional output of the network.

## DISCUSSION

In this work, we demonstrated the use of a split-intein protein-splicing strategy to reconstitute a TALE transcriptional activator from inactive parts, to generate AND logic circuits in mammalian cells. We first assayed six putative amino acid split sites in TAL118 and identified two that allowed intein-mediated splicing. The most efficient split site is located upstream of cysteine 490 within a protein loop region (Figure 1B), which is in accordance with our previous results using zinc finger proteins and suggests that protein secondary structures are critical for the efficiency of protein splicing (5). Given the strong conservation of TAL repeats, we expect that the identified split site will also work when using TALEs with different DNA binding domains (31). We used a TALE with a binding site computationally designed to be orthogonal to all promoters in the human and mouse genome to reduce the likelihood of interference with endogenous gene expression. Of note, the two engineered TALE sub-fragments are also unlikely to bind in the genome. Specifically, the N-terminal TAL118 fragment contains only 6 TAL repeats, which is below the threshold of 6.5 repeats needed for DNA binding (9). Whereas the DNA binding domain of the C-terminal TAL118 fragment includes more TAL repeats (13.5), our experiments revealed that it is not able to induce reporter expression by itself (Figure 1C). We speculate that a lack of repeat 0, which recognizes the conserved 5′ thymine found in almost all naturally occurring TALE recognition sites, prevents this fragment from binding to DNA (9,10,25). It is also possible that the fused intein domain itself inhibits DNA binding.

We also showed that three-piece intein splicing can be performed in mammalian cells. This finding allowed us to generate an AND circuit in mammalian cells that can integrate three positive signals. The two- and three-input TALE-based logic circuits presented here extend the toolbox for engineering synthetic genetic networks in mammalian cells.

TALE-based logic gates have both advantages and disadvantages compared with other approaches in biological computation. In comparison with RNA-based circuits, one disadvantage of TALE- and other TF-based logic computation is its relatively long response-time. On the other hand, whereas RNA-based logic is generally used to integrate combinations of small molecules (32) or miRNAs (33), transcriptional circuits make it possible to respond to the activity of a combination of endogenous promoters. This has, for example, been useful in building a TF-based AND gate circuit that integrates two cancer-cell-specific promoters (34). Our TALE-based AND gate circuit offers several advantages due to the flexibility of DNA binding sequences that TALEs provide. First, the flexibility in DNA binding makes it possible to build logic circuits that are orthogonal to each other and to the host genome. Second, by changing the sequence of the TALE, one could envision direct regulation of endogenous genes by spliced TALEs. Finally, fusing the split TALE to a repressor domain instead of a transcriptional activation domain could enable to repress specific genes in response to two or three concurrent signals, effectively performing NAND gate computation. By combining two repressors or activators, TALEs could also be used to build NOR and OR gates, respectively (5).

Currently, most genetic circuits in the field of mammalian synthetic biology are encoded on multiple plasmids and tested in transient transfections (5,33,35). As many applications may require stable maintenance in the cell type of interest, it is important to show that circuits are functional when integrated in the genome at a low copy. We here assembled relatively large genetic constructs and tested them after PiggyBac transposase-mediated low-copy genomic integration. This allowed us to demonstrate that a TALE-based logic circuit is functional in a genomic environment. Practical applications of bio-computational networks, for example in a clinical setting, may further necessitate their genomic integration into safe harbor sites (36). Along this line, we here used a method for site-directed single-copy integration in the genome of mouse ES cells (23). The system depends on negative selection and thereby circumvents the need of a resistance cassette that would increase construct size and might interfere with circuit function. In addition, this integration method makes it possible to compare the activity of different circuit variants in the same genomic environment. Taking advantage of this system, we compared variants of an AND circuit in the same genomic context and identified the promoter strength in the reporter as a critical parameter to enhance its transcriptional output. Our finding that TALE-based logic computation is functional after single-copy genomic integration in ES cells suggests that such circuits could be used as sensors in cell therapy applications or for directing *in vitro* differentiation in tissue engineering.

## SUPPLEMENTARY DATA

Supplementary Data are available at NAR Online.

## FUNDING

This work was supported by funds from a European Molecular Biology Organization Fellowship and a Human Frontier Science Program Fellowship (to F.L.); a National Science Foundation Graduate Research Fellowship awarded (to J.P.T.); a Natural Sciences and Engineering Research Council of Canada Postdoctoral Fellowship (to J.H.C.); a National Institutes of Health training grant [GM007598 to R.R.R.]; funds from NIH [R01GM036373]; and Defense Advanced Research Projects Agency (DARPA) [4500000572 to P.A.S.]. Funding for open access charge: NIH [R01GM036373].

*Conflict of interest statement*. None declared.

## Supplementary Material

Supplementary Data
