# Supplementary material for: Two- and three-input TALE-based AND logic computation in embryonic stem cells
Source: Nucleic Acids Res. 2013 Aug 27;41(21):9967–75. doi: 10.1093/nar/gkt758 (PMC3834826; doi:10.1093/nar/gkt758)
Supplement: Supplementary Data [file supp_41_21_9967__index.html]

Two- and three-input TALE-based AND logic computation in embryonic stem cells — Supplementary Data 

# Two- and three-input TALE-based AND logic computation in embryonic stem cells

## Supplementary Data

files

**Files in this Data Supplement:**

- Supplementary Data - docx file
- Supplementary Data - xlsx file
- Supplementary Data - xlsx file
- Supplementary Data - xlsx file
