# Supplementary material for: Two- and three-input TALE-based AND logic computation in embryonic stem cells
Source: Nucleic Acids Res. 2013 Aug 27;41(21):9967–75. doi: 10.1093/nar/gkt758 (PMC3834826; doi:10.1093/nar/gkt758)
Supplement: Supplementary Data [file supp_gkt758_nar-01693-h-2013-File006.docx]

**SUPPLEMENTARY INFORMATION**

**Two- and three-input TALE-based AND logic computation in embryonic stem cells**

Florian Lienert^1^, Joseph P Torella^1^, Jan-Hung Chen^1^, Michael Norsworthy^1^, Ryan R Richardson^1^ & Pamela A Silver^1,2^*

^1^ Department of Systems Biology, Harvard Medical School, Boston, MA 02115, USA

^2^ Wyss Institute for Biologically Inspired Engineering, Harvard University, Boston, MA 02115, USA

* To whom correspondence should be addressed: Tel.: + 1 617 432 6401, Fax: + 1 617 432 5012, Email: pamela_silver@hms.harvard.edu

**Table of Contents**

**Supplementary Figure S1. Example of raw data and overview of flow cytometry gating strategy.**

**Supplementary Figure S2. Testing of TAL118.**

**Table S1. Experimental DNA constructs and their constituent subparts**.

**Table S2. DNA plasmids co-transfected for each experiment.**

**Table S3. Flow cytometry data.**

**Supplementary Figure S1. Example of raw data and overview of flow cytometry gating strategy.** CFP vs. mCh scatter plot and histograms for transiently transfected AND gate circuit (blue dots and traces) and control circuit (red dots and traces). The scatter plot shows gating for mCh and CFP expression in live cells. To control for variations in transfection efficiency among different samples, we first gated for cells that are positive for mCh, which is expressed from a constitutive CMV promoter in all tested constructs (center histogram, orange gate). Within these mCh+ cells we determined the percentage of CFP positive cells (right histogram, green gate). In the scatter plot representation this number corresponds to the number of CFP+/mCh+ cells divided by the total number of mCh+ cells:

% CFP+ in mCh+ = Q2/(Q2+Q4)

mCh+ and CFP+ gates were determined on an untransfected control sample.

**Supplementary Figure S2. Testing of TAL118.** (**A**) Schematic of the plasmids used for testing reporter activation by TAL118. TAL118 is tagged with a self-cleaving t2A:mCherry fluorescent protein and expressed from a CMV promoter. The reporter plasmid expresses CFP under control of a HSV minimal promoter (m-pr) containing 1 to 6 binding sites for TAL118 (TAL118 BS). (**B**) Co-transfection of reporter plasmids with an off-target TALE (TAL248) or with TAL118. CFP activity was measured by flow cytometry and is reported as percentage of CFP+ in all mCh+ cells.

**Table S1. Experimental DNA constructs and their constituent subparts**. DNA constructs for testing AND circuits expressed from separate plasmids are listed with their constituent BioBrick subparts (DNA constructs A). DNA constructs for assembly of circuits on a single bacterial artificial chromosome (BAC) plasmid are listed with their constituent subparts (DNA constructs B). These DNA constructs B are flanked by homology regions (L1 to L5), which were used for assembling them onto two different BAC plasmids by isothermal assembly. See *TABLE_S1.xls*

**Table S2. DNA plasmids co-transfected for each experiment.** Plasmids and plasmid amounts transfected in transient transfection experiments. See *TABLE_S2.xls*

**Table S3. Flow cytometry data.** Flow cytometry data for transient transfection experiments and for stem cell colonies with stably integrated circuits. See *TABLE_S3.xls*
